# Association of the TyG–GGT index, a novel insulin resistance marker, with incident diabetes mellitus: a large-scale retrospective cohort study

**DOI:** 10.3389/fendo.2026.1735979

**Published:** 2026-04-21

**Authors:** Yajing Gao, Yuting Yan, Chuang Gao, Jiaqian Zhu, Yong Han

**Affiliations:** 1 Department of Anesthesiology, Shenzhen Maternity and Child Healthcare Hospital, Women and Children's Medical Center, Southern Medical University, Shenzhen, Guangdong, China; 2 Department of Emergency, Shenzhen Dapeng New District Kuichong People’s Hospital, Shenzhen, Guangdong, China; 3 Department of Neurology, Yiwu Central Hospital, Yiwu, Zhejiang, China; 4 Department of Neurology, Shenzhen University, Shenzhen Second People’s Hospital, The First Affiliated Hospital of Shenzhen University, Shenzhen, Guangdong, China; 5 Department of Emergency, Shenzhen Second People’s Hospital, The First Affiliated Hospital of Shenzhen University, Shenzhen, Guangdong, China

**Keywords:** diabetes mellitus, insulin resistance, nonlinear, predictive value, TyG-GGT index

## Abstract

**Objective:**

Research on the association between TyG-GGT index and diabetes mellitus (DM) risk remains scarce. This study aimed to investigate the relationship between TyG-GGT and DM incidence.

**Methods:**

This retrospective cohort investigation enrolled 8,678 participants who underwent comprehensive health screenings at Kuichong People’s Hospital in Shenzhen from 2018 through 2023. Cox proportional hazards regression models were employed to assess the association between TyG-GGT and DM risk, and Cox proportional hazards regression model with restricted cubic spline functions was used to evaluate non-linear relationships. Subgroup analyses and sensitivity analyses further verified the stability of these findings. Finally, receiver operating characteristic (ROC) curve methodology and time-dependent ROC analysis were performed to determine the predictive capacity of TyG-GGT for incident DM within a 5-year period.

**Results:**

Following multivariable adjustments, higher TyG-GGT levels were found to be associated with elevated DM risk, demonstrating an HR of 1.116 (95% CI: 1.041-1.196) per 50-unit increase in TyG-GGT. Additionally, a non-linear association between them was observed, exhibiting a threshold value at 380. When below this inflection point, the HR per 50-unit increase in TyG-GGT was 1.723 (95% CI: 1.500-1.979), while above this value the association was not statistically significant. Additionally, in predicting DM risk, TyG-GGT had the highest AUC value (0.732), while the AUC values of TG (0.635), GGT (0.649), FPG (0.660), and TyG (0.675) were all lower than this value. Time-dependent ROC analysis revealed that the AUC values of TyG-GGT remained stable between 0.7292-0.7338 over a prediction horizon of 1.0 to 5.0 years. The stability of these results was further corroborated via sensitivity analysis.

**Conclusion:**

This study found that TyG-GGT demonstrated an independent positive association and non-linear relationship with DM risk, with an inflection point at 380. TyG-GGT below 380 was associated with higher observed DM risk. Additionally, TyG-GGT exhibits discriminatory performance for DM risk assessment and may serve as a clinically useful predictor, thereby aiding clinicians in early identification of high-risk individuals and providing a novel perspective for optimizing clinical prevention and management of DM.

## Introduction

Diabetes mellitus (DM) is a complex metabolic disorder caused by the interaction of genetic and environmental factors, characterized by insulin deficiency, impaired insulin action, and glucose metabolism disorders (1). According to the International Diabetes Federation, the Diabetes Atlas reported that, in 2017, an estimated 425 million individuals were living with DM worldwide; by 2045, this figure is expected to rise to 629 million (2). Furthermore, DM can cause multiple complications, such as those affecting the renal, neurological, ocular, and cardiovascular system (3–6), and has become one of the leading causes of disability and death (7). With the continuously rising incidence of DM and the heavy burden of related mortality, it has become a major challenge facing global health systems (3, 8, 9). Therefore, identifying risk factors for DM and implementing targeted preventive interventions is of significant clinical importance for reducing the incidence of DM and its related complications.

Insulin resistance (IR) refers to a pathophysiological state characterized by diminished biological effects of insulin, serving as a common pathogenic mechanism underlying various metabolic disorders including non-alcoholic fatty liver disease (NAFLD), obesity, metabolic syndrome, and DM (10–13). Studies have demonstrated that the triglyceride-glucose (TyG) index, derived from the product of fasting plasma glucose (FPG) and triglyceride (TG), has been established as an effective and reproducible surrogate marker for assessing IR (14, 15). Furthermore, gamma-glutamyl transferase (GGT), as a significant biomarker reflecting hepatic fat accumulation and oxidative stress, exhibits elevated levels that are closely associated with IR and the development of DM (16–18). Consequently, a composite index integrating TyG, and GGT may more accurately reflect IR status compared with individual parameters.

In recent years, the triglyceride-glucose-gamma-glutamyl transferase(TyG-GGT) index, as a novel composite indicator integrating TyG and GGT, has demonstrated promising predictive capability for both NAFLD diagnosis and assessment of severe liver fibrosis (19, 20). However, no studies have yet investigated the association between TyG-GGT and DM. Given existing evidence indicating that DM and NAFLD share common pathophysiological mechanisms including inflammatory response, IR, and oxidative stress (21, 22). Therefore, we hypothesize that elevated TyG-GGT levels may be associated with increased DM risk. Accordingly, we conducted a retrospective cohort study to examine the relationship between TyG-GGT and DM in the Chinese adult population.

## Methods

### Study design and study population

This investigation adopted a retrospective cohort design. Participants were individuals who opted for routine medical check−ups at Kuichong People’s Hospital (Dapeng New District, Shenzhen) between January 2018 and December 2023. The observation window spanned 2018–2023, with baseline assessment conducted in 2018 and follow-up assessments performed through 2023, resulting in a maximum potential follow-up duration of 5 years for each participant. However, the actual follow-up duration varied across individuals based on the timing and frequency of their health examination visits. TyG−GGT was the independent variable, and DM was the dependent variable (coded in binary form: 0 for non−DM and 1 for DM).

**Figure 1:**
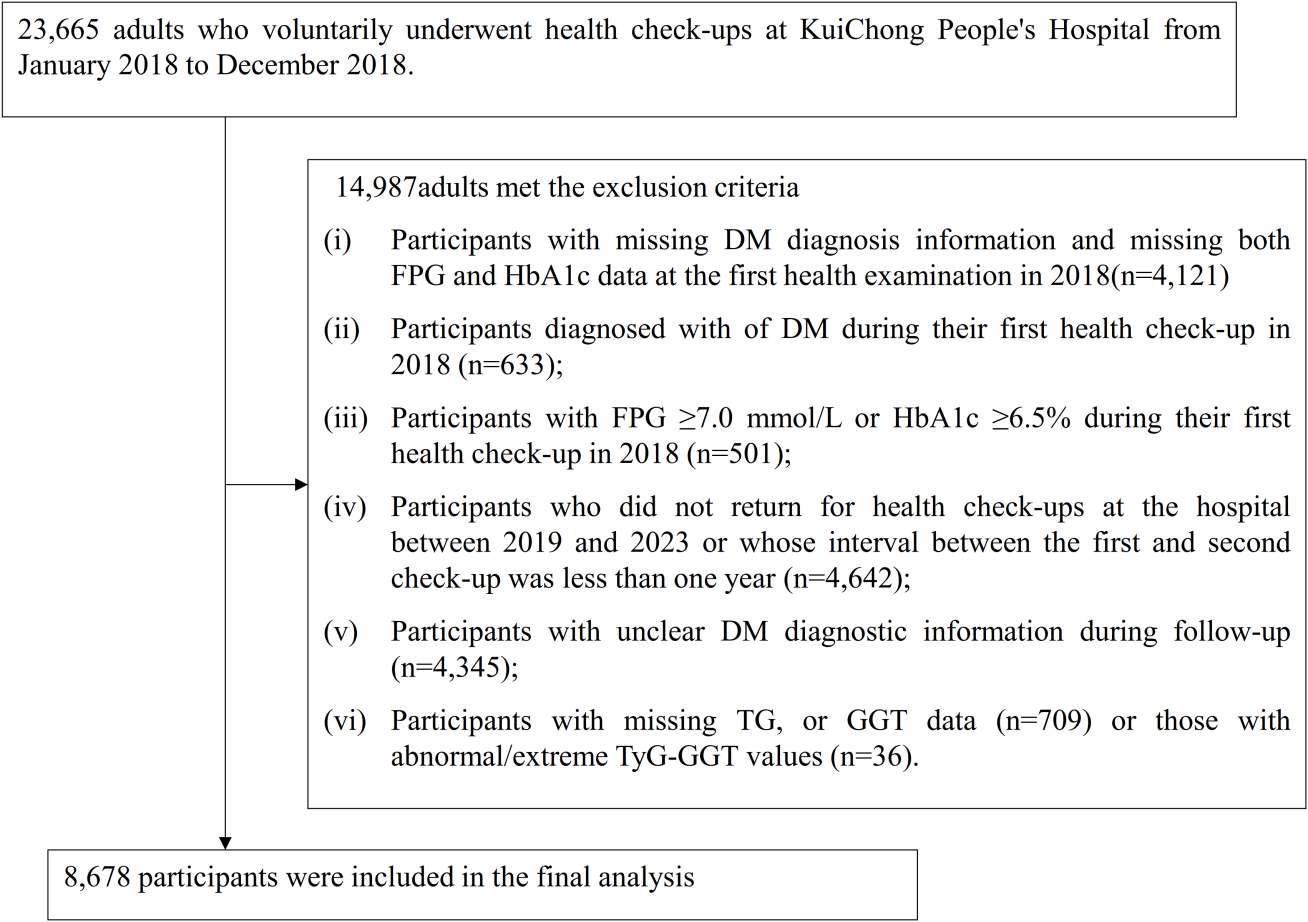
Flowchart illustrating the study participants.

The initial study population included 23,665 individuals older than 18 years who completed routine health examinations between January and December 2018. Participants were excluded based on the following conditions: (i)Participants with missing DM diagnosis information and missing both FPG and hemoglobin A1c (HbA1c) data at the first examination in 2018 (n = 4,300); (ii) Individuals identified as having DM during the initial health assessment conducted in 2018 (n=633); (iii) Those showing FPG values ≥7.0 mmol/L or HbA1c levels ≥6.5% at the baseline 2018 evaluation (n=501); (iv)Participants who did not return to the hospital for health examinations from 2019 to 2023 or had an interval of less than one year between the first and second examinations (n=4,642); (v) Individuals with indeterminate DM diagnosis throughout follow-up assessment (n=4,345); (vi)Participants with missing TG or GGT data (n=709) or abnormal/extreme TyG-GGT values (n=36). The final analytical cohort comprised 8,678 subjects. Subject enrollment procedures are illustrated in **Figure 1**.

### Ethical approval and consent

Ethical approval was obtained from the Ethics Committee at Kuichong People’s Hospital in Shenzhen’s Dapeng New District (reference: 2024005). Due to the retrospective design utilizing completely de-identified participant information, the Ethics Committee formally waived informed consent requirements. Furthermore, the research conduct complied rigorously with Declaration of Helsinki principles and followed all relevant ethical standards and regulatory mandates.

### Variables

#### TyG-GGT

The TyG-GGT index was calculated using the formula: Ln (TG × FPG ÷ 2) × GGT, where GGT was measured in U/L, and both FPG and TG were expressed in mg/dL. Note that the baseline measurements for FPG and TG were recorded in mmol/L in the clinical database. Prior to TyG-GGT calculation, these values were converted to mg/dL using the following conversion factors: FPG (mg/dL) = FPG (mmol/L) × 18; TG (mg/dL) = TG (mmol/L) × 88.57. This unit standardization was necessary to maintain consistency with the published TyG-GGT literature and to ensure accurate representation of the exposure variable across statistical analyses (20, 23).

#### Diagnosis and follow-up of DM

Incident DM was defined as participants who were free of DM at the 2018 baseline but reported DM during subsequent follow-up. Diagnostic criteria were met when any of the following conditions was satisfied: self-reported physician-diagnosed DM during follow-up, FPG ≥7.0 mmol/L, or HbA1c≥6.5% (24).

DM history information was collected through standardized questionnaires administered at each examination, which included whether participants had ever been diagnosed with DM by a physician, the date of diagnosis, and whether they had received glucose-lowering treatment. The date of DM onset was defined as the date of the health examination when participants first met the diagnostic criteria for DM (FPG ≥7.0 mmol/L or HbA1c ≥6.5%), or the date when the follow-up questionnaire identified that participants had been diagnosed with DM by a physician. Follow-up duration was calculated as the time between baseline (2018) and DM diagnosis date. Participants without DM diagnosis during follow-up were censored at their last clinical examination date.

### Covariates

Covariate selection was based on our clinical experience and findings from previous studies (16, 20). The covariates included in this study comprised: (i) continuous variables: low-density lipoprotein cholesterol (LDL-c),body mass index (BMI), HbA1c, total cholesterol (TC), high-density lipoprotein cholesterol (HDL-c), diastolic blood pressure (DBP),waist circumference (WC), aspartate aminotransferase (AST), C-reactive protein (CRP),alanine aminotransferase (ALT), serum creatinine (Scr), systolic blood pressure (SBP), and age; (ii) categorical variables: smoking status, drinking status, antihypertensive medication (HTN-MED), physical activity, dyslipidemia (DLP), antihyperlipidemic medication (DLP-MED), hypertension (HTN), and sex.

### Data collection

Certified clinical staff carried out anthropometric evaluations, and baseline characteristics were obtained through standardized questionnaires. Baseline information was obtained through standardized questionnaire administration. The questionnaires encompassed behavioral factors including tobacco use and alcohol intake, along with sociodemographic characteristics such as sex, age, and HTN. Blood pressure was measured at each examination using standard mercury sphygmomanometers. During each examination, venous specimens were drawn after a fast of at least 10 hours. Laboratory analyses of TC, TG, HDL-c, LDL-c, and FPG were assayed with a Beckman 5800 automated analyzer. Based on standardized questionnaire data, drinking status was defined as follows: Never drinkers: Individuals who never consumed alcohol or consumed fewer than 12 drinks per year; Current drinkers: Individuals who currently drink at any frequency; Ever drinkers: Individuals with a history of drinking, including former drinkers who have quit and those with intermittent drinking patterns.

### Handling of missing data

Missing data is a common and unavoidable situation in observational studies. In this analysis, multiple variables had missing values, including drinking status (533, 6.14%), smoking status (324, 3.73%), WC (9, 0.10%), DBP (17, 0.20%), SBP (17, 0.20%), DLP (119, 1.37%), Scr (159,1.83%), AST (182, 2.10%), and ALT (180, 2.10%). To minimize potential bias caused by missing data, we employed multiple imputations to fill missing values in the dataset (25, 26). This study employed multiple imputation by chained equations (MICE) with 10 iterations, and appropriate imputation algorithms were selected based on variable types. Continuous variables were imputed using predictive mean matching (PMM) to preserve the distribution characteristics of the original data and prevent imputed values from exceeding reasonable ranges. Categorical variables were imputed using logistic regression. Five imputed datasets were generated to allow independent analysis of estimates such as means and regression coefficients. Subsequently, Rubin’s rules were applied to combine the obtained estimates. Variables included in the imputation model were: SBP, Scr, TG, BMI, WC, TC, LDL-c, HDL-c, age, HbA1c, AST, ALT, CRP, GGT, sex, HTN, HTN-MED, DLP, DBP, DLP-MED, drinking status, smoking status, and physical activity. Missing values were assumed to be Missing at Random (MAR), following established analytical standards (26).

### Statistical analysis

To facilitate intergroup comparisons, baseline characteristics were stratified by quartiles of TyG-GGT. Continuous variables conforming to normality were presented as mean ± standard deviation (SD), while non-normally distributed data were described using median and interquartile range (IQR). Categorical variables were displayed as frequencies alongside their respective percentages. Between-group comparisons of continuous data utilized analysis of variance (ANOVA) or Kruskal-Wallis tests, whereas categorical variable comparisons employed chi-square analysis (χ²).

This study employed univariate and multivariate Cox proportional hazards regression models to examine the association between TyG-GGT and the risk of DM. Prior to model fitting, we assessed the proportional hazards (PH) assumption using Schoenfeld residuals testing. Both global and covariate-specific tests were conducted to verify the validity of the Cox proportional hazards model. Results demonstrated that neither the main exposure variable (TyG-GGT) nor global test violated the PH assumption, with all P values > 0.05. These findings support the use of Cox proportional hazards regression for our analyses. In addition, prior to model construction, variance inflation factors (VIFs) were calculated for all candidate covariates to assess multicollinearity, with VIF > 5 being used as the threshold to indicate significant collinearity. Three models were sequentially constructed: (i) Model I: unadjusted for any covariates; (ii) Model II: adjusted for age, BMI, and sex; (iii) Model III: adjusted for smoking status, BMI, HbA1c, physical activity, Scr, DLP-MED, ALT, DBP, HDL-c, age, LDL-c, HTN, AST, drinking status, and SBP. The adjusted variables were based on previous studies and clinical experience. Due to multicollinearity with other predictive factors, TC and WC were excluded from the multivariate models (**Supplementary Table 1**).

Cox proportional hazards regression models incorporating restricted cubic spline functions with 4 knots (positioned at the 10th, 33rd, 67th, and 90th percentiles of TyG-GGT distribution) were employed to explore possible nonlinear associations between TyG-GGT and DM risk. A recursive algorithm with grid search strategy was applied to determine the inflection point. Grid search was conducted across the entire range of TyG-GGT values at 1-unit intervals, and the log -likelihood ratio of the two-piecewise Cox regression model was calculated, aiming to minimize the P-value from the likelihood ratio test. Subsequently, segmented Cox regression analysis was performed on either side of the identified threshold, and the most appropriate model was selected via likelihood ratio testing.

Previous studies have established significant associations between HTN, obesity, smoking and glucose metabolism (27–29). To verify the robustness of our results, we conducted sensitivity analyses by sequentially excluding participants with BMI≥28 kg/m² (30), smokers, or those diagnosed with HTN. In addition, given that this study is a retrospective cohort study, DM diagnosis was detected through periodic health examinations rather than continuous clinical monitoring. Therefore, the exact date of DM onset could not be precisely determined, resulting in an interval-censoring problem. A sensitivity analysis was also conducted, in which the event time was redefined as “the midpoint between the date of the previous normal health examination and the date of the current abnormal health examination,” and the multivariable Cox regression analysis was rerun with the same adjustment variables. This approach provides a more conservative estimate of the true event date within this time interval. Furthermore, in the multivariable Cox regression models, we incorporated generalized additive models (GAM) to include continuous covariates as smoothed curves.Moreover, to assess the robustness of the imputation method, we conducted a complete case analysis as a sensitivity analysis using data before imputation, by excluding subjects with any missing values in the variables analyzed. Lastly, E-values were computed to evaluate possible effects of unmeasured and unknown confounding variables on the observed relationship linking TyG-GGT with DM risk. The E-value represents the minimum relative risk that an unmeasured confounder must have with both the exposure and outcome simultaneously to completely explain the observed association. Specifically, any unmeasured confounder must simultaneously satisfy the following two necessary conditions to completely explain the observed association (1): The relative risk of the unmeasured confounder with the exposure (RR_EU) exceeds the E-value; and (2) The relative risk of the unmeasured confounder with the outcome (DM) (RR_UD) exceeds the E-value (31, 32).

Subgroup analyses were carried out with stratified Cox regression model, using strata defined by sex, smoking, drinking, physical activity level, and categorized age, SBP, and DBP. Age was grouped as <30, 30–40, 40–50, and ≥50 years, while SBP was classified as <140 or ≥140 mmHg, and DBP as <90 or ≥90 mmHg, according to clinical thresholds (33). Covariate-adjusted models incorporated smoking, BMI, HbA1c, physical activity, Scr, DLP-MED, ALT, DBP, HDL-c, age, LDL-c, HTN, AST, drinking status, and SBP, while omitting stratification variables. We evaluated potential interaction terms by comparing models with and without these terms through likelihood ratio testing.

Finally, we assessed the predictive ability of TyG, FPG, TG, GGT, and TyG-GGT for DM risk by constructing receiver operating characteristic (ROC) curves. The corresponding area under the curve (AUC), best thresholds, sensitivity, and specificity were calculated. In addition, time-dependent ROC analysis was performed to assess the predictive performance of TyG-GGT for DM at different time points during the follow-up period (specifically at 1.0, 2.0, 3.0, 4.0, and 5.0 years). The AUC, optimal threshold, sensitivity, and specificity were also subsequently calculated.

Reporting of the results adhered to the STROBE statement guidelines (34). All statistical analyses were performed using R software (v3.4.3) and Empower software (v4.2). Statistical significance was defined as two-sided P values < 0.05.

## Results

### Participant characteristics

**Figure 2:**
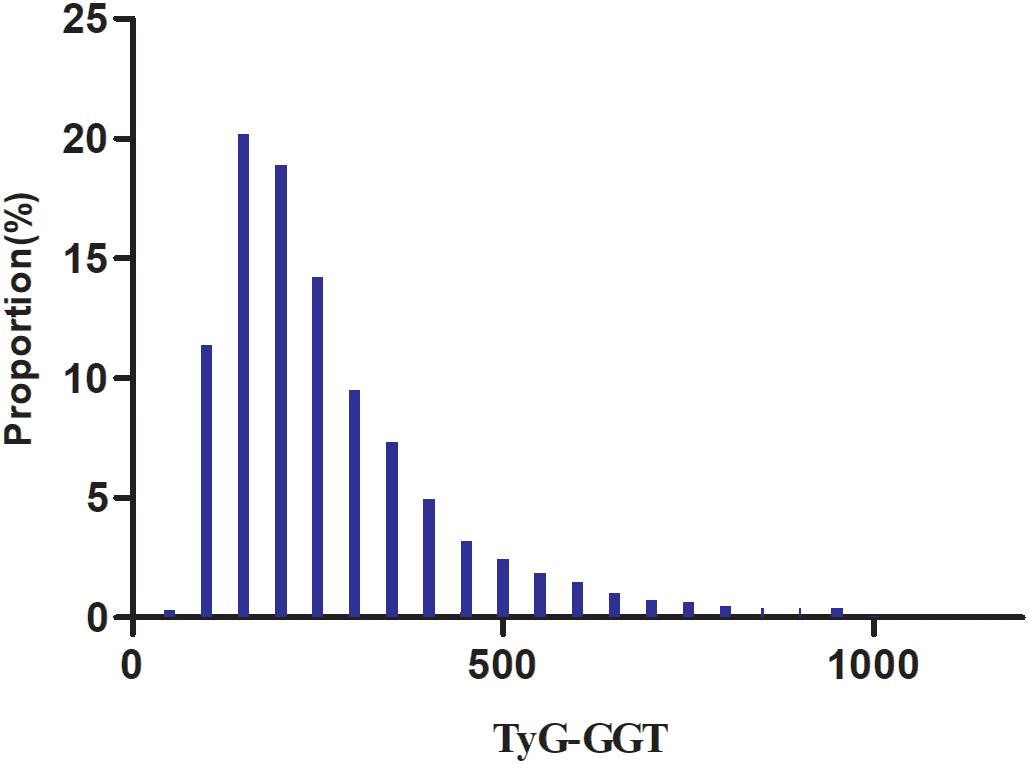
Distribution of TyG-GGT. The distribution appeared skewed, spanning from 42.87 to 995.38, with a median (IQR) of 222.08 (185.57-327.83).

Demographic and clinical characteristics for the 8,678 study participants are displayed in **Table 1**, where male participants comprised 74.52% of the total population. TyG-GGT distribution exhibited skewed patterns, spanning 42.87 to 995.38, with median (IQR) values of 222.08 (185.57-327.83) as illustrated in **Figure 2**. Participants were stratified into four groups based on TyG-GGT quartiles: Q1(<158.55), Q2 (158.55-222.07), Q3(222.07-327.80), and Q4(≥327.80). Compared with the Q1 group, participants in higher quartile groups had higher levels of age, SBP, BMI, TG, DBP, Scr, WC, TC, ALT, TyG, FPG, HbA1c, AST, LDL-c, CRP, and GGT, while HDL-c levels were relatively lower. Additionally, higher quartile groups showed higher proportions of males, HTN, DLP, HTN-MED, DLP-MED, smokers, sedentary individuals, and alcohol consumers compared with the Q1 group.

**Table 1:** Baseline characteristics of participants.

TyG-GGT quartile	Q1 (<158.55)	Q2 (158.55-222.07)	Q3 (222.07-327.80)	Q4 (≥327.80)	P-value
N	2170	2168	2170	2170	
Age(years)	39.49 ± 8.18	41.87 ± 8.73	42.94 ± 8.79	42.84 ± 8.28	<0.001
SBP (mmHg)	110.55 ± 11.27	116.23 ± 11.07	119.53 ± 11.92	121.45 ± 13.08	<0.001
DBP (mmHg)	71.81 ± 7.52	75.48 ± 7.32	77.76 ± 7.57	79.21 ± 8.08	<0.001
Scr (μmol/L)	69.07 ± 15.39	70.04 ± 15.24	69.92 ± 15.42	70.58 ± 15.82	0.019
BMI (kg/m^2^)	23.90 ± 3.03	25.46 ± 3.36	26.93 ± 3.76	27.99 ± 4.04	<0.001
WC (cm)	82.78 ± 10.41	89.44 ± 10.71	94.69 ± 11.03	97.64 ± 11.09	<0.001
**TG** (mmol/L)	0.89 (0.70-1.19)	1.11 (0.85-1.50)	1.38 (1.02-1.87)	1.69 (1.23-2.31)	<0.001
**HDL-c**(mmol/L)	1.44 ± 0.35	1.29 ± 0.34	1.19 ± 0.30	1.14 ± 0.29	<0.001
**LDL-c**(mmol/L)	2.82 ± 0.75	3.16 ± 0.84	3.31 ± 0.86	3.43 ± 0.90	<0.001
**TC** (mmol/L)	4.73 ± 0.81	5.02 ± 0.91	5.19 ± 0.94	5.44 ± 0.98	<0.001
TyG	8.12 ± 0.41	8.37 ± 0.43	8.60 ± 0.46	8.82 ± 0.51	<0.001
FPG (mmol/L)	4.61 ± 0.41	4.78 ± 0.44	4.92 ± 0.49	4.99 ± 0.49	<0.001
HbA1C (%)	4.53 ± 0.26	4.63 ± 0.28	4.72 ± 0.31	4.77 ± 0.31	<0.001
AST(U/L)	24.78 ± 11.26	27.58 ± 12.45	29.71 ± 7.83	34.06 ± 10.92	<0.001
ALT(U/L)	28.00 ± 10.30	34.21 ± 13.03	41.23 ± 15.14	52.93 ± 24.60	<0.001
CRP (mg/dL)	2.07 ± 5.16	2.22 ± 5.68	2.37 ± 4.35	2.86 ± 4.81	<0.001
GGT(U/L)	15.30 ± 2.67	22.68 ± 2.36	31.29 ± 3.62	55.23 ± 17.21	<0.001
Sex (n, %)					<0.001
Female	1296 (59.72%)	529 (24.40%)	228 (10.51%)	158 (7.28%)	
male	874 (40.28%)	1639 (75.60%)	1942 (89.49%)	2012 (92.72%)	
HTN (n, %)	91 (4.19%)	176 (8.12%)	254 (11.71%)	347 (15.99%)	<0.001
DLP (n, %)	356 (16.41%)	531 (24.49%)	681 (31.38%)	797 (36.73%)	<0.001
HTN-MED (n, %)	88 (4.06%)	178 (8.21%)	243 (11.20%)	352 (16.22%)	<0.001
DLP-MED (n, %)	89 (4.10%)	154 (7.10%)	230 (10.60%)	284 (13.09%)	<0.001
Smoking (n, %)	136 (6.27%)	161 (7.43%)	173 (7.97%)	200 (9.22%)	0.003
Physical Activity (n, %)					<0.001
Sedentary	387 (17.83%)	398 (18.36%)	461 (21.24%)	611 (28.16%)	
Light activity	831 (38.29%)	798 (36.81%)	821 (37.83%)	835 (38.48%)	
Moderate activity	736 (33.92%)	772 (35.61%)	692 (31.89%)	577 (26.59%)	
Vigorous activity	216 (9.95%)	200 (9.23%)	196 (9.03%)	147 (6.77%)	
Drinking status (n, %)					<0.001
Never drinkers	193 (9.94%)	157(7.91%)	137 (6.22%)	111 (4.36%)	
Current drinkers	250 (12.87%)	306 (15.42%)	379 (17.20%)	451 (17.70%)	
Ever drinkers	1499 (77.19%)	1521 (76.67%)	1688 (76.58%)	1986 (77.94%)	

Continuous variables were presented either as mean ± standard deviation or as median with interquartile range, depending on data distribution. Categorical data were reported as counts and percentages. Waist circumference (WC), Hemoglobin A1c(HbA1c), aspartate aminotransferase (AST), antihyperlipidemic medication (DLP-MED), total cholesterol (TC), serum creatinine (Scr), high-density lipoprotein cholesterol (HDL-c), gamma-glutamyl transferase(GGT), diastolic blood pressure (DBP), Fasting Plasma Glucose(FPG), body mass index (BMI), hypertension (HTN), C-reactive protein (CRP), Triglycerides(TG), Systolic blood pressure (SBP), dyslipidemia (DLP), low-density lipoprotein cholesterol (LDL-c), antihypertensive medication (HTN-MED), Triglyceride-glucose index (TyG), alanine aminotransferase (ALT).

### The incidence of DM

Following a median follow-up time of 1.89 years [interquartile range (IQR): 1.34–3.42 years], incident DM developed in 310 participants (3.57% of the 8,678 total) who were normoglycemic at baseline. The distribution of follow-up duration was as follows: 4,591 participants (52.9%) had 1–2 years of follow-up, 1,754 participants (20.21%) had 2–3 years, 1,258 participants (14.5%) had 3–4 years, and 1,075 participants (12.39%) had >4 years of follow-up. Stratified by TyG-GGT quartiles, the DM incidence rates per 10,000 person-years were 107.67, 166.62,318.21, and 467.11, respectively. The overall cumulative DM incidence was 3.57%, with specific incidence rates by quartile as follows: Q1 1.47%, Q2 2.26%, Q3 4.29%, and Q4 6.27%. Notably, compared with participants in Q1 (lowest TyG-GGT levels), those in Q4 (highest TyG-GGT levels) showed significantly higher DM incidence (P for trend <0.001) (**Table 2**).

**Table 2:** Incidence rate of diabetes (% or per 10000 person-year).

TyG-GGT	Participants (n)	DM events (n)	Incidence rate (95% CI) (%)	Per 10000 person-year
Total	8678	310	3.57(3.18 -3.96)	238.66
Q1	2170	32	1.47 (0.97-1.98)	107.67
Q2	2168	49	2.26 (1.63-2.89)	166.62
Q3	2170	93	4.29 (3.43-5.14)	318.21
Q4	2170	136	6.27 (5.25-7.29)	467.11
P for trend			<0.001	

**Figure 3:**
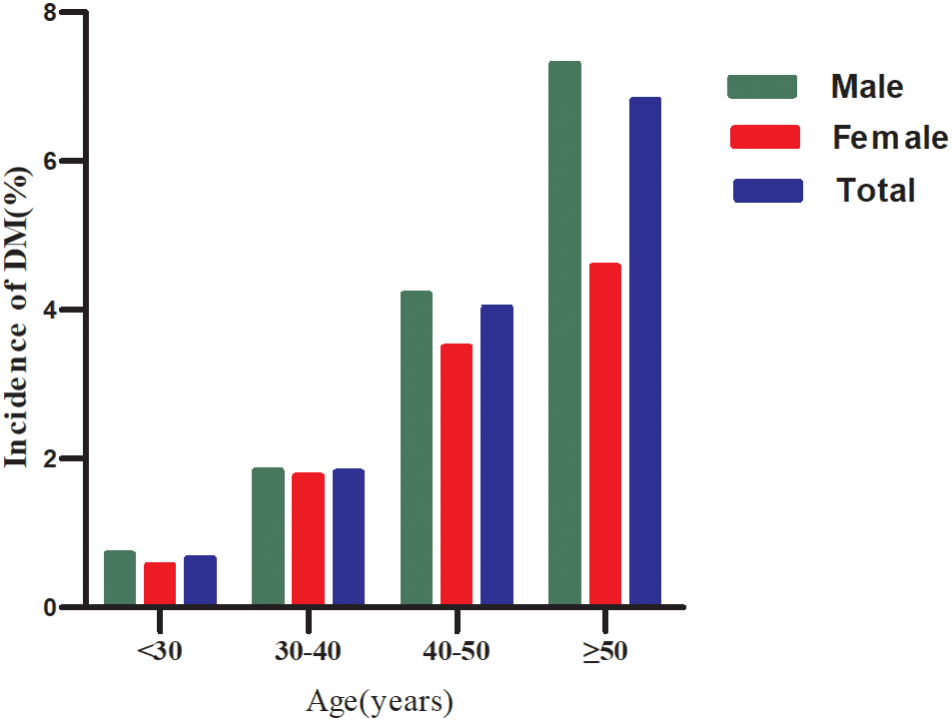
Diabetes incidence (%) stratified by age and sex.

Age stratification was performed according to the following age groups: <30 years, 30–40 years, 40–50 years, and ≥50 years. Males in all age groups showed higher DM risk compared to females (**Figure 3**). Additionally, DM incidence increased with age in both males and females.

**Figure 4:**
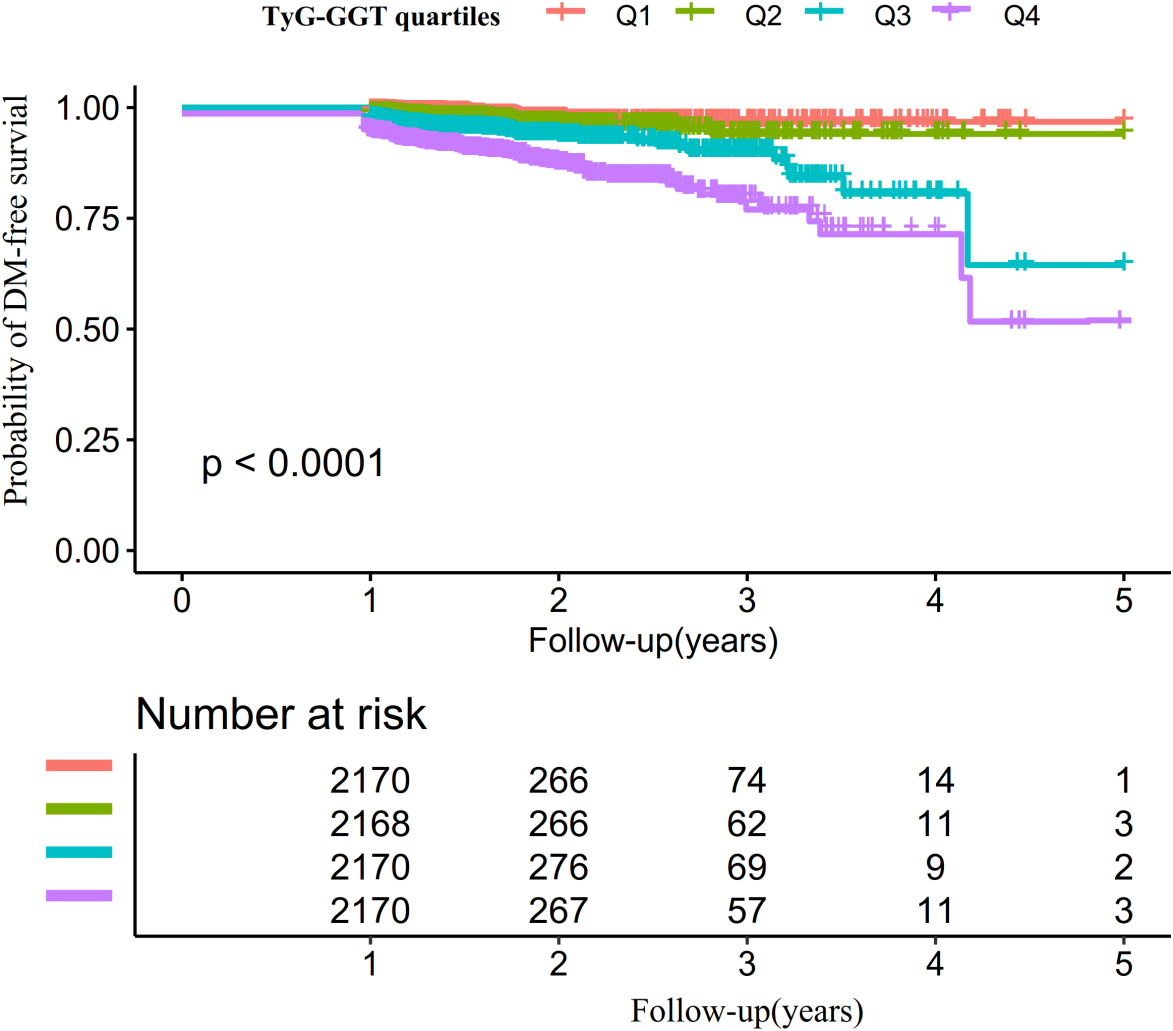
Kaplan-Meier curves illustrating DM–free survival probabilities across TyG-GGT quartiles.

Kaplan-Meier survival curves stratified by TyG-GGT quartiles (**Figure 4**) displayed the probabilities of DM-free survival. Notable disparities in survival without DM emerged across TyG-GGT quartile groups (log-rank test, P < 0.001). Analysis demonstrated that subjects in the uppermost TyG-GGT quartile exhibited the greatest DM risk.

### The relationship between TyG-GGT and the risk of DM

Three Cox proportional hazards regression models were constructed to investigate the relationship between TyG-GGT and DM risk. Univariate Model I demonstrated a 27.1% increased risk of DM per 50-unit elevation in TyG-GGT (HR = 1.271; 95% CI: 1.208-1.337). Following adjustment for sex, age, and BMI in Model II, a 50-unit increment in TyG-GGT yielded an 18.5% elevated DM risk (HR = 1.185; 95% CI: 1.117-1.258). The fully adjusted Model III demonstrated that this relationship remained significant: a 50-unit TyG-GGT increase conferred an 11.6% higher DM risk(HR = 1.116; 95% CI: 1.041-1.196) (**Table 3**).

**Table 3:** Association of TyG-GGT with DM risk using various models.

Exposure	Model I (HR,95%CI) P	Model II (HR,95%CI) P	Model III (HR,95%CI) P	Model IV (HR,95%CI) P
TyG-GGT (per 50-unit)	1.271 (1.208, 1.337) <0.001	1.185 (1.117, 1.258) <0.001	1.116 (1.041, 1.196) 0.002	1.108 (1.032, 1.189) 0.005
TyG-GGT quartile				
Q1	Ref	Ref	Ref	Ref
Q2	1.553 (1.004, 2.424) 0.043	1.264 (0.797, 2.002) 0.319	1.137 (0.713, 1.813) 0.591	1.095 (0.679, 1.766) 0.708
Q3	3.018 (2.019, 4.510) <0.001	1.974 (1.270, 3.067) 0.003	1.440 (1.009, 2.280) 0.005	1.323 (1.121, 2.132) 0.001
Q4	4.443 (3.023, 6.530) <0.001	2.594 (1.677, 4.011) <0.001	1.762 (1.109, 2.799) 0.016	1.596 (1.085, 2.586) 0.038
P for trend	<0.001	<0.001	0.004	0.019

CI, confidence, Ref, reference; HR, Hazard ratios.

Model I, we did not adjust other covariates.

Model II, we adjust sex, age and BMI.

Model III, we adjust age, BMI, drinking status, ALT, HDL-c, LDL-c, HbA1c, physical activity, DBP, smoking status, Scr, AST, HTN, DLP-MED, and SBP.

Model IV, we adjusted age(smooth), BMI (smooth), drinking status, ALT (smooth), HDL-c(smooth), LDL-c(smooth), HbA1c (smooth), physical activity, DBP (smooth), smoking status, Scr (smooth), AST (smooth), HTN, DLP-MED, and SBP (smooth).

HR, hazard ratio; Ref, reference; CI, confidence.

Furthermore, after stratification by TyG-GGT quartiles, the data were re-entered into the Cox proportional hazards regression model. With Q1 serving as the referent category, multivariable-adjusted analysis revealed HRs of 1.137 (95% CI: 0.713-1.813) for Q2, 1.440 (95% CI: 1.009-2.280) for Q3, and 1.762 (95% CI: 1.109-2.799) for Q4. These results indicated that compared with Q1, Q2 showed a 13.7% increased risk of DM, while Q3 and Q4 showed 44% and 76.2% increased risks, respectively (**Table 3**, Model III).

### Sensitivity analysis

To assess the stability of the study results, various sensitivity analyses were conducted. Initially, GAM was employed to incorporate continuous covariates as smoothed curves into the model. The results obtained from this approach (**Table 3**, Model IV) were largely consistent with those from the fully adjusted Model III. Specifically, for every 50-unit elevation in TyG-GGT, the risk of DM increased by 10.8% (HR = 1.108; 95% CI: 1.032-1.189). In addition, sensitivity analysis confined to participants with BMI under 28 kg/m², the connection between TyG-GGT (for each 50-unit upsurge) and DM risk was still evident after correcting for confounders (HR = 1.132; 95% CI: 1.086-1.286). After excluding participants with HTN, the results were consistent: each 50-unit increase in TyG-GGT yielded an HR of 1.116 (95% CI: 1.026-1.213) for DM. Finally, among non-smokers, this association remained significant, with an HR of 1.100 (95% CI: 1.024-1.181) (**Table 4**).

**Table 4:** Association of TyG-GGT with DM risk across various sensitivity analyses.

Exposure	Model I (HR,95%CI) P	Model II (HR,95%CI) P	Model III (HR,95%CI) P
TyG-GGT (per 50-unit)	1.132 (1.086, 1.286) 0.007	1.116 (1.026, 1.213) 0.010	1.100 (1.024, 1.181) 0.009
TyG-GGT quartile			
Q1	Ref	Ref	Ref
Q2	1.545 (0.833, 2.864) 0.167	1.002 (0.601, 1.672) 0.993	1.151 (0.715, 1.853) 0.562
Q3	2.339 (1.241, 4.408) 0.009	1.434 (0.870, 2.363) 0.157	1.432 (1.094, 2.293) 0.014
Q4	2.286 (1.137, 4.597) 0.020	1.871 (1.128, 3.104) 0.015	1.743 (1.086, 2.796) 0.021
P for trend	0.011	0.002	0.006

CI, confidence; Ref, reference; HR, Hazard ratios;.

Model I was a sensitivity analysis among subjects having BMI values under 28 kg/m² (n=6,044). Age, BMI, ALT, HDL-c, LDL-c, HbA1c, physical activity, DBP, Scr, smoking, hypertension, AST, drinking status, DLP_MED, and SBP were adjusted.

Model II was a sensitivity analysis following the exclusion of HTN individuals (N = 7,810). Age, BMI, drinking status, ALT, HDL-c, LDL-c, HbA1c, smoking, DBP, Scr, AST, physical activity, DLP_MED, and SBP were adjusted.

Model III was a sensitivity analysis following the exclusion of smokers (N = 8,111). Age, BMI, ALT, HDL-c, LDL-c, HbA1c, physical activity, DBP, Scr, hypertension, AST, drinking status, DLP_MED, and SBP were adjusted.

Additionally, a sensitivity analysis was also conducted, in which the event time was redefined as “the midpoint between the date of the previous normal health examination and the date of the current abnormal health examination,” and the multivariable Cox regression analysis was rerun. A more conservative estimate of the true event date within this time interval was thereby obtained. The association between TyG-GGT (per 50-unit increase) and DM risk was found to be significant, with an HR of 1.113(95% CI: 1.038, 1.194), which was basically consistent with the main analysis results (**Supplementary Table 4** Model I). Furthermore, to assess the robustness of our findings to unmeasured confounding, we conducted an E-value sensitivity analysis. For the primary association between TyG-GGT and DM (adjusted HR = 1.116 per 50-unit increase, 95% CI: 1.041–1.196), the calculated E-value was 1.38. The E-value corresponding to the lower limit of the 95%CI was 1.25. In this study, although the relative risk of unmeasured confounding with DM (RR_UD = 1.69) exceeded the E-value threshold (1.38), its relative risk with TyG-GGT exposure (RR_EU = 1.34) remained below this threshold. Therefore, unmeasured confounding cannot simultaneously satisfy both necessary conditions and cannot entirely explain our findings through its combined association with both the exposure and outcome. Moreover, in the complete case sensitivity analysis, all participants with missing values in any covariate were excluded, and the analysis was re-evaluated. The results showed that TyG-GGT (per 50-unit increase) was associated with an HR of 1.103 (95%CI: 1.024, 1.189) for DM, which was generally consistent with the results from the multiple imputation analysis (**Supplementary Table 4** Model II). These sensitivity assessments strengthened the credibility and robustness of our findings.

### Non-linear relationship between TyG-GGT and the risk of DM

**Figure 5:**
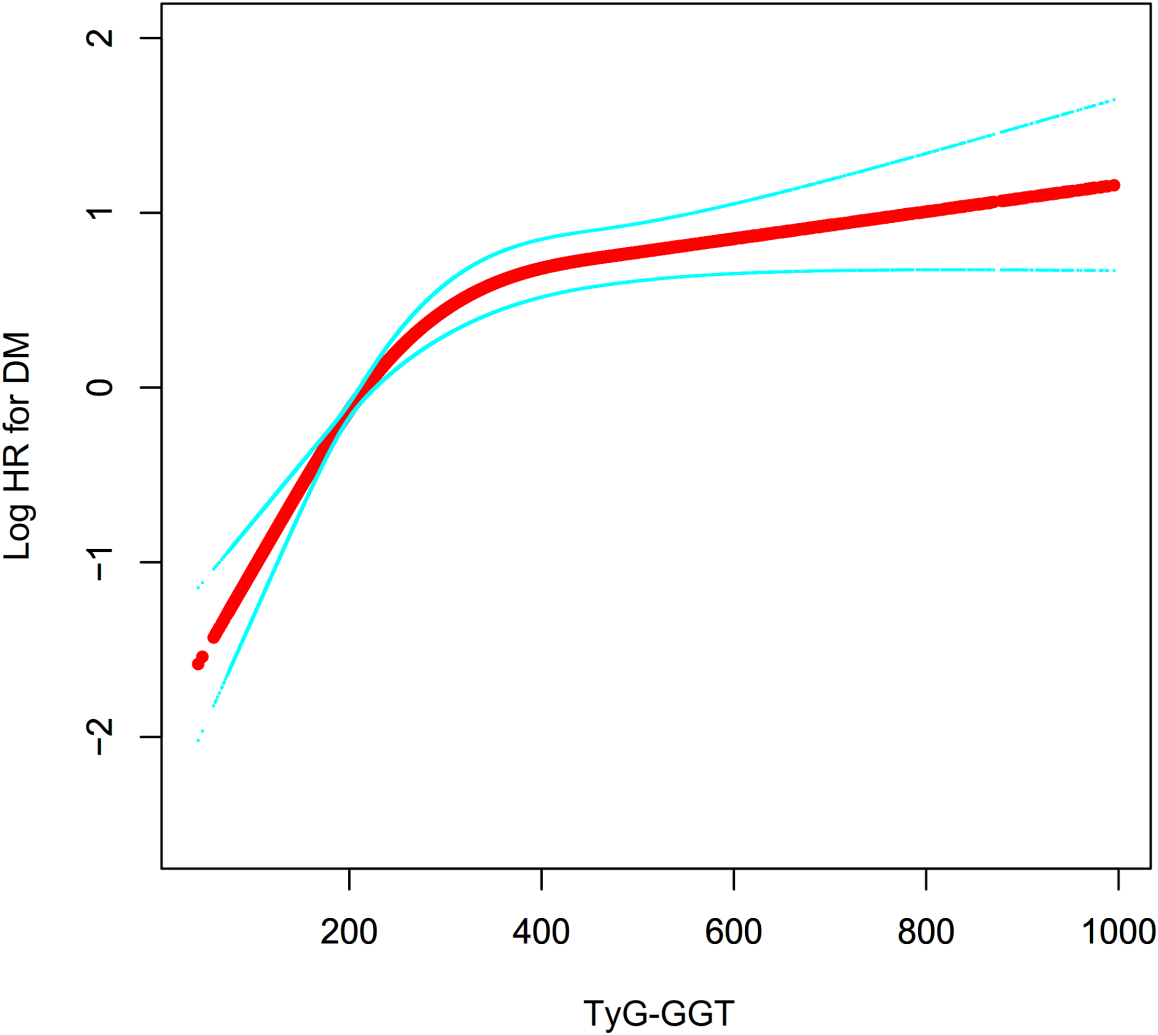
Nonlinear relationship between TyG-GGT and the risk of DM.

Using restricted cubic splines function in the Cox hazards regression model revealed a nonlinear association between TyG-GGT and DM risk (P for nonlinearity <0.001; **Figure 5**). A recursive algorithm with grid search strategy identified an inflection point at a TyG-GGT value of 380. Bootstrap resampling analysis (500 iterations) confirmed threshold stability, with a 95% CI of (366–402). Segmented Cox regression model was employed for calculating HRs with CIs on either side of this threshold. At TyG-GGT levels below this cutpoint, the HR relating TyG-GGT to DM risk reached 1.723 (95% CI: 1.500-1.979). In contrast, at values exceeding the threshold, the HR measured 1.051 (95% CI: 0.949-1.164), showing no statistical significance (**Table 5**).

**Table 5:** The result of two-piecewise Cox regression.

Incident DM:	HR(95%CI) P-value
Inflection points of TyG-GGT	380 (366–402)
<380(per 50-unit)	1.723 (1.500, 1.979) <0.001
≥380(per 50-unit)	1.051 (0.949, 1.164) 0.342
P for log-likelihood ratio test	<0.001

CI, confidence;, Ref, reference; HR, Hazard ratios.

we adjust age, BMI, drinking status, ALT, HDL-c, LDL-c, HbA1C, physical activity, DBP, smoking status, Scr, AST, hypertension, DLP-MED, and SBP.

### Subgroup analysis

Across predefined and exploratory subgroup evaluations (**Supplementary Table 2**), we observed no significant interaction effects between TyG-GGT and multiple covariates, including sex, physical activity, age, drinking, SBP, smoking, and DBP(all P for interaction >0.05). These findings indicated that these factors did not significantly influence or modify the association between TyG-GGT and DM risk.

### ROC analysis of the predictive value of TyG, FPG, TG, GGT, and TyG-GGT for DM risk

**Figure 6:**
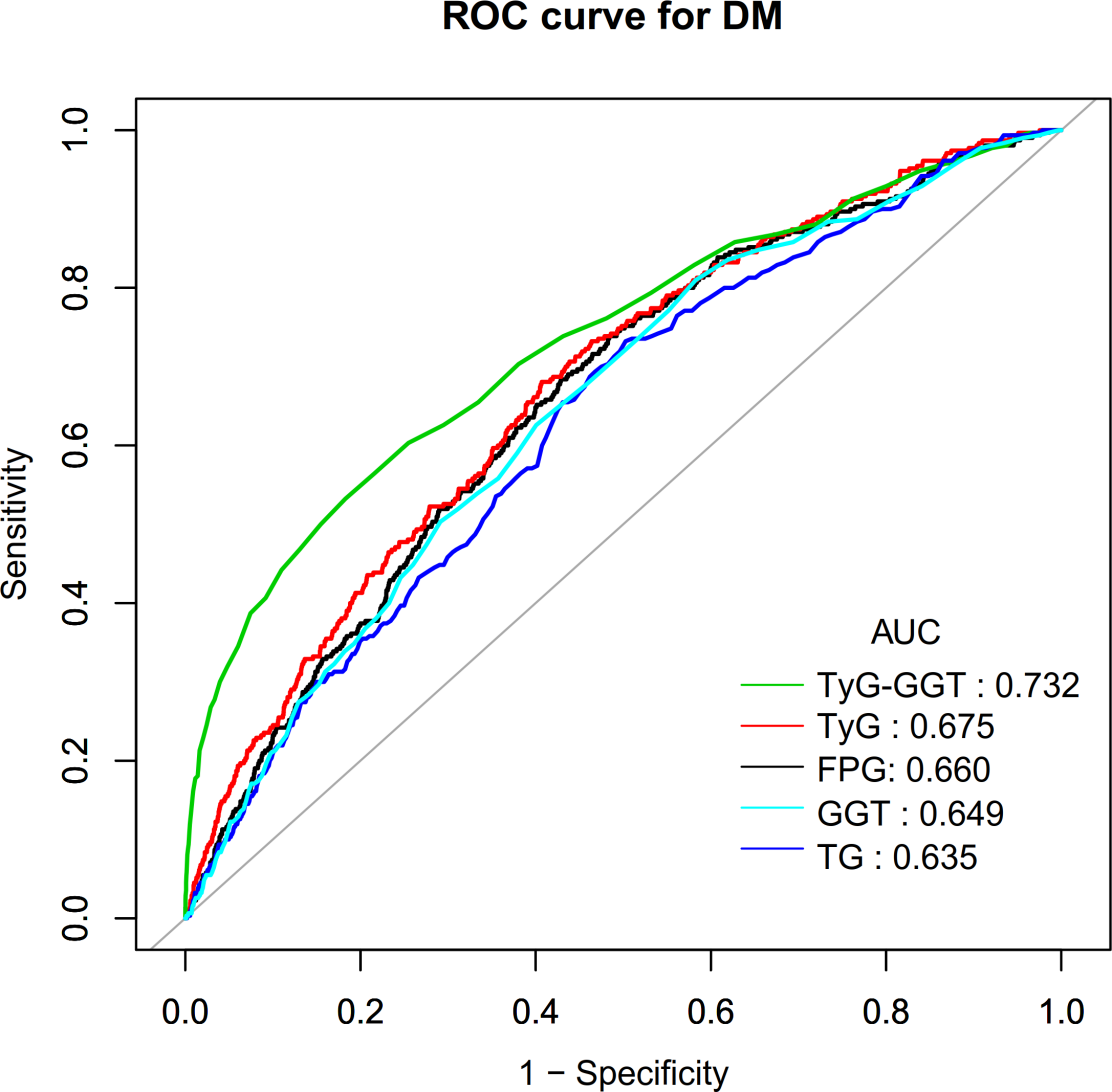
ROC curves for TyG-GGT, FPG, TyG, and GGT prediction of DM.

ROC curves were plotted to evaluate the predictive ability of TyG, FPG, GGT, TG, and TyG-GGT for DM risk within a 5-year period (**Figure 6**). The AUC values for each variable were as follows: TG: 0.6352 < GGT: 0.649 < FPG: 0.660 < TyG: 0.675 < TyG-GGT: 0.732. The Youden index values for FPG, TyG, TyG-GGT, TG, and GGT were 0.2552, 0.2737, 0.3496, 0.2297, and 0.2272, respectively, with corresponding best cut-off values of 5.1944, 8.5732, 405.86, 106.5000, and 23.5000 (**Table 6**). This indicated that Compared with FPG, TyG, TG, and GGT, TyG-GGT displayed superior discriminatory ability, demonstrating incremental predictive value. Nonetheless, these findings should be considered exploratory and not interpreted as evidence of strong predictive ability.

**Table 6:** The predictive value of TyG-GGT, TG, FPG, TyG, and GGT for DM risk.

Test	AUC (95%CI)	Best threshold	Specificity	Sensitivity	Yueden index
FPG (mmol/L)	0.6604 (0.6307-0.6901)	5.19	0.5713	0.6839	0.2552
TyG	0.6752 (0.6455-0.7048)	8.57	0.5931	0.6806	0.2737
TyG-GGT	0.7318 (0.7000-0.7635)	405.86	0.8173	0.5323	0.3496
TG (mmol/L)	0.6352 (0.6048-0.6655)	1.20	0.4974	0.7323	0.2297
GGT(U/L)	0.6490 (0.6190-0.6790)	23.50	0.4175	0.8097	0.2272

FPG, Fasting Plasma Glucose; TG, Triglycerides; GGT, Gamma-Glutamyl Transferase; TyG, Triglyceride-glucose index.

### Time-dependent ROC analysis of the predictive value of TyG-GGT for DM risk

To further assess the predictive ability of TyG-GGT for DM risk across different time intervals, we performed time-dependent ROC analysis. The results showed that the AUC values of TyG-GGT for predicting DM incidence at 1.0, 2.0, 3.0, 4.0, and 5.0 years were 0.7338 (95% CI: 0.6935-0.7741), 0.7292 (95% CI: 0.6952-0.7631), 0.7321 (95% CI: 0.6997-0.7646), 0.7342 (95% CI: 0.7024-0.7660), and 0.7318 (95% CI: 0.7000-0.7635), respectively. These results indicated that TyG-GGT demonstrated relatively stable predictive value for DM development across both short-term and long-term follow-up periods (**Supplementary Figure 1**, **Supplementary Table 5**). Additionally, calibration curve analysis of the predictive value of TyG-GGT for DM risk within 5 years demonstrated acceptable concordance between observed DM risk and predicted DM risk throughout the entire 5-year follow-up period (**Supplementary Figure 2**).

## Discussion

This study demonstrated that TyG-GGT exhibited an independent positive association with DM risk. Additionally, a saturation effect curve was observed with an inflection point at a TyG-GGT value of 380, with the relationship exhibiting differences on both sides of this inflection point. Additionally, ROC curve analysis showed that TyG-GGT outperformed conventional TG, FPG, TyG, and GGT in predicting DM risk.

Multiple studies have confirmed that the TyG index serves as an effective and reproducible composite indicator of IR, with elevated levels closely associated with increased DM risk (35–37). A large-scale cohort study involving 201,298 participants demonstrated that the TyG index was an independent risk factor for DM after multivariate adjustment (HR = 3.34, 95% CI: 3.11-3.60) (35). A separate cohort investigation comprising 15,012 prediabetic Chinese adults demonstrated that individuals in the uppermost TyG index quartile exhibited a 1.03-fold elevated risk of DM relative to those in the lowest quartile (HR = 2.03, 95% CI: 1.71–2.40) (37). Additionally, a cohort study drawing upon China Health and Retirement Longitudinal Study (CHARLS) data, which included 6,258 individuals ≥45 years old, also established an important positive association between TyG index and DM risk (HR = 1.75, 95% CI: 1.56-1.97) (36).

Furthermore, GGT, as a sensitive biomarker of hepatic lipid accumulation and oxidative stress, can mediate the occurrence of IR through mechanisms such as inducing chronic inflammation and oxidative stress (38, 39). Studies have shown that GGT levels are closely associated with DM risk (16, 17). A cohort study containing 346,206 participants showed that after adjusting for confounding factors, compared with those with normal GGT levels, the HR for DM risk in women with high GGT levels was 3.05 (95%CI: 2.73-3.41) and in men was 2.60 (95%CI: 2.47-2.73) (16). Another cohort study including 15,464 Japanese adults using multivariable Cox proportional hazards model analysis showed that compared with the lowest GGT quintile, the highest quintile had an 83% increased risk of DM (HR = 1.83, 95%CI: 1.06-3.15) (17).

Recently, a composite indicator formed by the TyG index and GGT, namely TyG-GGT, has been proposed and demonstrates strong predictive ability in NAFLD diagnosis and severe hepatic fibrosis assessment (19, 20). In addition, previous studies suggest that NAFLD is closely connected to DM (40). Encompassing 33 investigations involving 501,022 participants, a meta-analytical review revealed that NAFLD patients exhibited 1.19-times elevated DM risk relative to non-NAFLD individuals (HR: 2.19, 95% CI: 1.93-2.48) (41).Therefore, based on the above evidence, we hypothesize that TyG-GGT may be closely associated with DM risk. Regrettably, no studies to date have investigated the relationship between TyG-GGT and DM risk. This study verified our hypothesis that elevated TyG-GGT levels are positively linked with DM risk. Additionally, we treated TyG-GGT as both categorical and continuous variables to minimize information loss and more precisely quantify its association with outcomes. To further ensure the reliability of results, we performed sensitivity analyses in participants with BMI <28 kg/m², no HTN, and never smokers, which further confirmed the consistency of these findings in these specific subgroups. In conclusion, elucidating the relationship between TyG-GGT and DM risk has important clinical significance. Incorporating TyG-GGT into routine clinical assessment may help clinicians optimize risk stratification and management. Increased physical activity and improved dietary patterns, which were also associated with lower TG and FPG levels, may be relevant strategies for lower DM incidence and potentially reduced public health burden.

Moreover, exploratory ROC analysis revealed that TyG-GGT exhibited higher AUC and Youden index than any of its individual components (including FPG, GGT, TyG, and TG). Additionally, Time-dependent ROC analysis revealed that the AUC values of TyG-GGT remained stable between 0.7292-0.7338 over a prediction horizon of 1.0 to 5.0 years, indicating relatively consistent predictive performance for both short- and long-term DM incidence. Therefore, as a novel, clinically accessible, and reproducible composite index, TyG-GGT may serve as an effective predictor for incident DM, thereby assisting in early identification of high-risk populations and providing valuable insights for early intervention and treatment. Future prospective studies with external validation in independent populations are essential to confirm the predictive utility of TyG-GGT for clinical risk stratification.

The specific mechanism underlying the association between TyG-GGT and DM onset remains incompletely understood, but it may be related to IR. Research confirms that IR is a key determinant of DM development and progression (13). As an important biomarker reflecting hepatobiliary function, elevated GGT levels may lead to IR through mechanisms including activation of oxidative stress responses, induction of chronic inflammation, and promotion of hepatic steatosis (18, 38, 39, 42). Studies demonstrate that elevated GGT levels are closely associated with DM risk (16, 17). Additionally, the TyG index serves as an effective surrogate marker for assessing IR (14). Therefore, the mechanism underlying the association between TyG-GGT and DM risk may be related to the relationships between TyG and GGT with IR.

Furthermore, participants were stratified based on TyG-GGT quartiles, and the results of the multivariable-adjusted model showed that compared with Q1, the HRs for DM risk in Q2, Q3, and Q4 were 1.137, 1.440, and 1.762, respectively. This indicates that the HR increase from Q1 to Q2 tends to be flat, while Q3 and Q4 show a significant upward trend, suggesting that TyG-GGT may have a nonlinear association with DM risk. To verify this hypothesis, we employed a Cox proportional hazards regression model with restricted cubic splines function, and the results revealed a nonlinear relationship between them, with an inflection point TyG-GGT at 380. When TyG-GGT was below 380, each 50-unit increase in TyG-GGT was associated with a 72.3% increase in DM risk; when TyG-GGT exceeded 380, this association was no longer statistically significant. Further analysis revealed that compared with the TyG-GGT<380 group, subjects with TyG-GGT≥380 had significantly higher levels of age, SBP, DBP, BMI, WC, TC, LDL-c, HbA1c, AST, ALT, and CRP, as well as higher proportions of HTN, DLP, smoking, sedentary lifestyle, and light activity (see **Supplementary Table 3**). These factors are closely associated with DM risk (27–29, 43, 44). In the cohort with TyG-GGT values below 380, the levels of these risk factors were lower, and the impact of TyG-GGT on DM risk was relatively stronger. Conversely, when TyG-GGT exceeded 380, the impact of TyG-GGT on DM risk became relatively weaker due to the presence of these risk factors. This might elucidate the curvilinear association linking TyG-GGT levels to DM risk. Compared with individuals with higher TyG-GGT levels, individuals with TyG-GGT below 380 demonstrated a stronger association between TyG-GGT and DM risk. Whether lifestyle factors such as dietary adjustments and increased physical activity could influence TyG-GGT levels and subsequently DM risk warrants further investigation in prospective and intervention studies.

While these findings regarding the nonlinear relationship and inflection point are noteworthy, the relatively short median follow-up time of 1.89 years should be considered when interpreting these results. The follow-up period is relatively short for studying incident DM in a low-risk screening population and may underestimate long-term DM risk. Nevertheless, the detection of significant associations despite the low event rate (3.57% cumulative incidence) suggests the robustness of the TyG-GGT-DM relationship. Longer-term prospective cohort follow-up would be essential to (1): confirm the stability of the identified nonlinear association and inflection point at TyG-GGT = 380 during extended surveillance (2); more accurately estimate long-term DM incidence trajectories; and (3) determine whether the threshold effect and risk stratification pattern identified in the current observation period persist or shift with prolonged follow-up. The inflection point at 380 and the distinctly different risk patterns on either side represent preliminary findings that may provide insights for early prevention strategies but require validation through larger-scale prospective cohort studies with extended follow-up duration (≥5–10 years).

The present study has several important strengths: (i) Based on current literature, this is the first study to explore the association between TyG-GGT and DM risk. The study analyzed TyG-GGT both as a continuous variable and as a categorical variable, minimizing information loss and thereby providing a more comprehensive and accurate assessment of its association with DM risk. (ii) This study revealed a non-linear relationship between TyG-GGT and DM risk and identified the inflection point, which represents an important advancement. (iv) Multiple imputation was employed to handle missing data, significantly improving statistical power and reducing bias caused by missing covariate information. (v) To confirm the robustness of our results, several sensitivity analyses were performed, encompassing: converting TyG-GGT into a categorical variable; incorporating continuous covariates as curves in GAMs; estimation of E-values for quantifying potential bias from unobserved and unknown confounders; and reassessment of the association between these variables following exclusion of subjects with obesity (BMI≥28 kg/m²), HTN, or smokers.

Nevertheless, certain constraints of our investigation merit acknowledgment. First, the study population was restricted to Chinese participants. This limitation constrains the external validity of the findings across different ethnic groups or geographic regions, necessitating validation in more heterogeneous populations. Second, only baseline measurements of TyG-GGT and other related parameters were collected, without evaluating the longitudinal changes in TyG-GGT over time. Therefore, future studies need to collect more comprehensive longitudinal data on TyG-GGT. Third, due to the retrospective cohort design based on periodic health examinations (rather than continuous monitoring), there is an interval-censoring problem in DM diagnosis. The exact time of DM onset cannot be precisely determined, as it may have occurred at any time between the previous normal examination and the subsequent abnormal examination. We conducted a sensitivity analysis using the midpoint between two consecutive health examinations as the DM event date, which provides a more conservative estimate of the true event date within this time interval. This was basically consistent with the main analysis results, demonstrating that our findings possess a certain degree of robustness. In the future, we will conduct prospective cohort studies with more frequent follow-ups, which will help determine more precise DM event dates and further validate our findings. Additionally, due to the retrospective cohort design, there were limitations in adjusting for potential confounding factors such as dietary habits, and insulin levels. Although we calculated the E-value and determined that these unmeasured confounders do not significantly affect the association between TyG-GGT and DM, this is merely a statistical explanation and cannot completely rule out its potential impact on the results. In the future, prospective cohort studies should be conducted to collect more confounding factors to further verify these findings. Lastly, we underscore that this cohort analysis established an independent association between TyG-GGT and DM development, yet cannot determine causal relationship.

## Conclusion

This study found an independent positive association and non-linear relationship between TyG-GGT and DM risk. Notably, when TyG-GGT was below 380, each 50-unit increase was associated with a 72.3% increase in DM risk. TyG-GGT, as a simple and easily accessible hematological indicator, has certain predictive value for DM risk. TyG-GGT may serve as a reliable biomarker offering new insights into optimizing clinical DM prevention and management strategies, warranting further investigation.

## Data Availability

The raw data supporting the conclusions of this article will be made available by the authors, without undue reservation.

## References

[1] Kuzuya T . Early diagnosis, early treatment and the new diagnostic criteria of diabetes mellitus. Br J Nutr. (2000) 84:S177–81. doi: 10.1079/096582197388644. PMID: 11242465

[2] Cho NH Shaw JE Karuranga S Huang Y Da Rocha Fernandes JD Ohlrogge AW . IDF Diabetes Atlas: Global estimates of diabetes prevalence for 2017 and projections for 2045. Diabetes Res Clin Pract. (2018) 138:271–81. doi: 10.1016/j.diabres.2018.02.023. PMID: 29496507

[3] Fradkin JE Cowie CC Hanlon MC Rodgers GP . Celebrating 30 years of research accomplishments of the diabetes control and complications trial/epidemiology of diabetes interventions and complications study. Diabetes. (2013) 62:3963–67. doi: 10.2337/db13-1108. PMID: 24264393 PMC3837062

[4] Preis SR Hwang S Coady S Pencina MJ D’Agostino RBS Savage PJ . Trends in all-cause and cardiovascular disease mortality among women and men with and without diabetes mellitus in the Framingham Heart Study, 1950 to 2005. Circulation. (2009) 119:1728–35. doi: 10.1161/CIRCULATIONAHA.108.829176. PMID: 19307472 PMC2789419

[5] Tandon N Ali MK Narayan KMV . Pharmacologic prevention of microvascular and macrovascular complications in diabetes mellitus: implications of the results of recent clinical trials in type 2 diabetes. Am J Cardiovasc Drugs. (2012) 12:7–22. doi: 10.2165/11594650-000000000-00000. PMID: 22217193

[6] Zhu J Hu Z Luo Y Liu Y Luo W Du X . Diabetic peripheral neuropathy: pathogenetic mechanisms and treatment. Front Endocrinol (Lausanne). (2023) 14:1265372. doi: 10.3389/fendo.2023.1265372. PMID: 38264279 PMC10803883

[7] Global, regional, and national comparative risk assessment of 84 behavioural, environmental and occupational, and metabolic risks or clusters of risks for 195 countries and territories, 1990-2017: a systematic analysis for the Global Burden of Disease Study 2017. Lancet. (2018) 392:1923–94. doi: 10.1016/S0140-6736(18)32225-6. PMID: 30496105 PMC6227755

[8] Sun H Saeedi P Karuranga S Pinkepank M Ogurtsova K Duncan BB . IDF Diabetes Atlas: Global, regional and country-level diabetes prevalence estimates for 2021 and projections for 2045. Diabetes Res Clin Pract. (2022) 183:109119. doi: 10.1016/j.diabres.2021.109119. PMID: 34879977 PMC11057359

[9] The global burden of cancer attributable to risk factors, 2010-19: a systematic analysis for the Global Burden of Disease Study 2019. Lancet. (2022) 400:563–91. doi: 10.1016/S0140-6736(22)01438-6. PMID: 35988567 PMC9395583

[10] McCracken E Monaghan M Sreenivasan S . Pathophysiology of the metabolic syndrome. Clin Dermatol. (2018) 36:14–20. doi: 10.1016/j.clindermatol.2017.09.004. PMID: 29241747

[11] Barazzoni R Gortan Cappellari G Ragni M Nisoli E . Insulin resistance in obesity: an overview of fundamental alterations. Eat Weight Disord. (2018) 23:149–57. doi: 10.1007/s40519-018-0481-6. PMID: 29397563

[12] Buzzetti E Pinzani M Tsochatzis EA . The multiple-hit pathogenesis of non-alcoholic fatty liver disease (NAFLD). Metabolism. (2016) 65:1038–48. doi: 10.1016/j.metabol.2015.12.012. PMID: 26823198

[13] Stumvoll M Goldstein BJ van Haeften TW . Type 2 diabetes: principles of pathogenesis and therapy. Lancet. (2005) 365:1333–46. doi: 10.1016/S0140-6736(05)61032-X. PMID: 15823385

[14] Guerrero-Romero F Simental-Mendía LE González-Ortiz M Martínez-Abundis E Ramos-Zavala MG Hernández-González SO . The product of triglycerides and glucose, a simple measure of insulin sensitivity. Comparison with the euglycemic-hyperinsulinemic clamp. J Clin Endocrinol Metab. (2010) 95:3347–51. doi: 10.1210/jc.2010-0288. PMID: 20484475

[15] Ramdas Nayak VK Satheesh P Shenoy MT Kalra S . Triglyceride Glucose (TyG) Index: A surrogate biomarker of insulin resistance. J Pak Med Assoc. (2022) 72:986–88. doi: 10.47391/JPMA.22-63. PMID: 35713073

[16] Park J Han K Kim H Cho J Yoon K Kim MK . Cumulative exposure to high γ-glutamyl transferase level and risk of diabetes: A nationwide population-based study. Endocrinol Metab (Seoul). (2022) 37:272–80. doi: 10.3803/EnM.2022.1416. PMID: 35413781 PMC9081297

[17] Zhao W Tong J Liu J Liu J Li J Cao Y . The dose-response relationship between gamma-glutamyl transferase and risk of diabetes mellitus using publicly available data: A longitudinal study in Japan. Int J Endocrinol. (2020) 2020:5356498. doi: 10.1155/2020/5356498. PMID: 32215009 PMC7054786

[18] Thamer C Tschritter O Haap M Shirkavand F Machann J Fritsche A . Elevated serum GGT concentrations predict reduced insulin sensitivity and increased intrahepatic lipids. Horm Metab Res. (2005) 37:246–51. doi: 10.1055/s-2005-861411. PMID: 15952086

[19] Rivière B Jaussent A Macioce V Faure S Builles N Lefebvre P . The triglycerides and glucose (TyG) index: A new marker associated with nonalcoholic steatohepatitis (NASH) in obese patients. Diabetes Metab. (2022) 48:101345. doi: 10.1016/j.diabet.2022.101345. PMID: 35339664

[20] Jin L Gu J Zhang Z Du C Xu F Huang X . TyG-GGT is a reliable non-invasive predictor of advanced liver fibrosis in overweight or obese individuals. Obes Surg. (2024) 34:1333–42. doi: 10.1007/s11695-024-07139-y. PMID: 38427150

[21] Tilg H Moschen AR Roden M . NAFLD and diabetes mellitus. Nat Rev Gastroenterol Hepatol. (2017) 14:32–42. doi: 10.1038/nrgastro.2016.147. PMID: 27729660

[22] Firneisz G . Non-alcoholic fatty liver disease and type 2 diabetes mellitus: the liver disease of our age? World J Gastroenterol. (2014) 20:9072–89. 25083080 10.3748/wjg.v20.i27.9072 PMC4112878

[23] Wang L Cong H Zhang J Hu Y Wei A Zhang Y . Triglyceride-glucose index predicts adverse cardiovascular events in patients with diabetes and acute coronary syndrome. Cardiovasc Diabetol. (2020) 19:80. doi: 10.1186/s12933-020-01054-z. PMID: 32534586 PMC7293784

[24] Diabetes advocacy: standards of care in diabetes-2024. Diabetes Care. (2024) 47:S307–08. doi: 10.2337/dc24-S017. PMID: 38078588 PMC10725796

[25] Groenwold RHH White IR Donders ART Carpenter JR Altman DG Moons KGM . Missing covariate data in clinical research: when and when not to use the missing-indicator method for analysis. Cmaj. (2012) 184:1265–69. doi: 10.1503/cmaj.110977. PMID: 22371511 PMC3414599

[26] White IR Royston P Wood AM . Multiple imputation using chained equations: Issues and guidance for practice. Stat Med. (2011) 30:377–99. doi: 10.1002/sim.4067. PMID: 21225900

[27] Guo Z Liu L Yu F Cai Y Wang J Gao Y . The causal association between body mass index and type 2 diabetes mellitus-evidence based on regression discontinuity design. Diabetes Metab Res Rev. (2021) 37:e3455. doi: 10.1002/dmrr.3455. PMID: 33860627

[28] Ohishi M . Hypertension with diabetes mellitus: physiology and pathology. Hypertens Res. (2018) 41:389–93. doi: 10.1038/s41440-018-0034-4. PMID: 29556093

[29] Durlach V Vergès B Al-Salameh A Bahougne T Benzerouk F Berlin I . Smoking and diabetes interplay: A comprehensive review and joint statement. Diabetes Metab. (2022) 48:101370. doi: 10.1016/j.diabet.2022.101370. PMID: 35779852

[30] Zhou B . Predictive values of body mass index and waist circumference for risk factors of certain related diseases in Chinese adults--study on optimal cut-off points of body mass index and waist circumference in Chinese adults. BioMed Environ Sci. (2002) 15:83–96. doi: 10.1046/j.1440-6047.11.s8.9.x. PMID: 12046553

[31] Haneuse S VanderWeele TJ Arterburn D . Using the E-value to assess the potential effect of unmeasured confounding in observational studies. Jama. (2019) 321:602–03. doi: 10.1001/jama.2018.21554. PMID: 30676631

[32] Gaster T Eggertsen CM Støvring H Ehrenstein V Petersen I . Quantifying the impact of unmeasured confounding in observational studies with the E value. BMJ Med. (2023) 2:e366. doi: 10.1136/bmjmed-2022-000366. PMID: 37159620 PMC10163534

[33] Program NHBP . The seventh report of the joint national committee on prevention, detection, evaluation, and treatment of high blood pressure. Bethesda (MD: National Heart, Lung, and Blood Institute (US (2004). 20821851

[34] von Elm E Altman DG Egger M Pocock SJ Gøtzsche PC Vandenbroucke JP . The Strengthening the Reporting of Observational Studies in Epidemiology (STROBE) Statement: guidelines for reporting observational studies. Int J Surg. (2014) 12:1495–99. doi: 10.1016/j.ijsu.2014.07.013. PMID: 25046131

[35] Li X Li G Cheng T Liu J Song G Ma H . Association between triglyceride-glucose index and risk of incident diabetes: a secondary analysis based on a Chinese cohort study: TyG index and incident diabetes. Lipids Health Dis. (2020) 19:236. doi: 10.1186/s12944-020-01403-7. PMID: 33161902 PMC7649000

[36] Li X Sun M Yang Y Yao N Yan S Wang L . Predictive effect of triglyceride glucose-related parameters, obesity indices, and lipid ratios for diabetes in a chinese population: A prospective cohort study. Front Endocrinol (Lausanne). (2022) 13:862919. doi: 10.3389/fendo.2022.862919. PMID: 35432185 PMC9007200

[37] Chen B Zeng J Fan M You Q Wang C Wang K . A longitudinal study on the impact of the TyG Index and TG/HDL-C ratio on the risk of type 2 diabetes in Chinese patients with prediabetes. Lipids Health Dis. (2024) 23:262. doi: 10.1186/s12944-024-02239-1. PMID: 39175004 PMC11340070

[38] Castellano I Merlino A . γ-Glutamyltranspeptidases: sequence, structure, biochemical properties, and biotechnological applications. Cell Mol Life Sci. (2012) 69:3381–94. doi: 10.1007/s00018-012-0988-3. PMID: 22527720 PMC11115026

[39] Corti A Belcastro E Dominici S Maellaro E Pompella A . The dark side of gamma-glutamyltransferase (GGT): Pathogenic effects of an ‘antioxidant’ enzyme. Free Radic Biol Med. (2020) 160:807–19. doi: 10.1016/j.freeradbiomed.2020.09.005. PMID: 32916278

[40] Zheng X Cao C He Y Wang X Wu J Hu H . Association between nonalcoholic fatty liver disease and incident diabetes mellitus among Japanese: a retrospective cohort study using propensity score matching. Lipids Health Dis. (2021) 20:59. doi: 10.1186/s12944-021-01485-x. PMID: 34130693 PMC8207755

[41] Mantovani A Petracca G Beatrice G Tilg H Byrne CD Targher G . Non-alcoholic fatty liver disease and risk of incident diabetes mellitus: an updated meta-analysis of 501–022 adult individuals. Gut. (2021) 70:962–69. doi: 10.1136/gutjnl-2020-322572. PMID: 32938692

[42] Whitfield JB . Gamma glutamyl transferase. Crit Rev Clin Lab Sci. (2001) 38:263–355. doi: 10.1080/20014091084227. PMID: 11563810

[43] Ceriello A Prattichizzo F . Variability of risk factors and diabetes complications. Cardiovasc Diabetol. (2021) 20:101. doi: 10.1186/s12933-021-01289-4. PMID: 33962641 PMC8106175

[44] The prevention of diabetes mellitus. Jama. (2021) 325:190. doi: 10.1001/jama.2020.17738. PMID: 33433568

