# Supplementary material for: Association of the TyG–GGT index, a novel insulin resistance marker, with incident diabetes mellitus: a large-scale retrospective cohort study
Source: Front Endocrinol (Lausanne). 2026 Apr 21;17:1735979. doi: 10.3389/fendo.2026.1735979 (PMC13138955; doi:10.3389/fendo.2026.1735979)
Supplement: Supplementary file 1 [file DataSheet1.docx]

**Association of the TyG–GGT index, a novel insulin resistance marker, with incident diabetes mellitus: a large-scale retrospective cohort study**

**Running title: TyG‑****GGT and DM**

**YaJing Gao^1#^, YuTing Yan^1#^, Chuang Gao^2^_,_ Jiaqian Zhu^3,4^**^*^**, Yong Han^5^**^*^

^1^Department of Anesthesiology, Affiliated Shenzhen Maternity and Child Healthcare Hospital, Southern Medical University, Shenzhen, 518028, China.

^2^ Department of Emergency, Shenzhen Dapeng New District Kuichong People's Hospital, Shenzhen 518000, Guangdong Province, China.

^3^Department of Neurology, Yiwu Central Hospital, 699 Jiangdong Road, Yiwu, 322000, Zhejiang, China.

^4^Department of Neurology, Shenzhen University, Shenzhen Second People's Hospital, The First Affiliated Hospital of Shenzhen University, Shenzhen 518035, Guangdong Province, China.

^5^Department of Emergency, Shenzhen Second People's Hospital, The First Affiliated Hospital of Shenzhen University, Shenzhen 518035, Guangdong Province, China.

**YaJing Gao^1#^and YuTing Yan^1#^**contributed equally.

***Correspondence:**

Jiaqian Zhu

Department of Neurology, The First Affiliated Hospital of Shenzhen University

3002 Sungang West Road, Futian District,

Shenzhen 518035,

Guangdong Province,

China.

Email: zhujiaqian1122@163.com

***Corresponding author**

Yong Han

Department of Emergency, Shenzhen Second People's Hospital

No.3002 Sungang West Road, Futian District,

Shenzhen 518035,

Guangdong Province,

China.

Email: [Hanyong511023@163.com](mailto:Hanyong511023@163.com)

**Table S1 collinearity screening**

|  | Step 1 | Step 2 | Step 3 | Step 4 |
| --- | --- | --- | --- | --- |
| TyG-GGT | 273.4 | 1.6 | 1.6 | 1.6 |
| Sex | 2.1 | 2 | 2 | 1.7 |
| Age | 1.3 | 1.3 | 1.3 | 1.3 |
| HTN | 4.5 | 4.5 | 4.5 | 4.5 |
| DLP | 1.3 | 1.3 | 1.3 | 1.3 |
| HTN-MED | 4.5 | 4.5 | 4.5 | 4.5 |
| DLP-MED | 1.3 | 1.3 | 1.3 | 1.3 |
| SBP | 3.2 | 3.2 | 3.2 | 3.2 |
| DBP | 3.1 | 3.1 | 3.1 | 3.1 |
| BMI | 4.3 | 4.3 | 4.3 | 1.7 |
| WC | 5.3 | 5.3 | 5.3 | NA |
| Smoking | 1 | 1 | 1 | 1 |
| TC | 47.8 | 41.1 | NA | NA |
| LDL-c | 41 | 35.7 | 1.2 | 1.2 |
| HDL-c | 7.6 | 6.4 | 1.6 | 1.6 |
| TG | 6.6 | 6.4 | 1.4 | 1.4 |
| HABIC | 1.4 | 1.3 | 1.3 | 1.3 |
| AST | 1.8 | 1.8 | 1.8 | 1.8 |
| ALT | 2.4 | 2.4 | 2.4 | 2.4 |
| CRP | 1 | 1 | 1 | 1 |
| Physical Activity | 1.1 | 1.1 | 1.1 | 1.1 |

**Variables excluded from collinearity screening: TC and WC**

**Table S2. Stratified associations between TyG-GGT (per 50-unit) and DM by age, sex, SBP, DBP, physical activity, smoking, and drinking.**

| Characteristic | No of participants | HR (95%CI) P value P for interaction |
| --- | --- | --- |
| Age(years) |  | 0.2938 |
| <30 | 453 | 1.259 (0.738, 2.147) 0.3985 |
| 30-40 | 3244 | 1.079 (0.942, 1.236) 0.2730 |
| 40-50 | 3294 | 1.069 (0.969, 1.180) 0.1823 |
| ≥50 | 1687 | 1.211 (1.093, 1.342) 0.0003 |
| Sex |  | 0.2611 |
| Male | 6467 | 1.131 (1.052, 1.216) 0.0009 |
| Female | 2211 | 1.016 (0.843, 1.224) 0.8677 |
| SBP (mmHg) |  | 0.1333 |
| <140 | 8060 | 1.094 (1.014, 1.181) 0.0197 |
| ≥140 | 618 | 1.240 (1.068, 1.439) 0.0047 |
| DBP (mmHg) |  | 0.5522 |
| <90 | 7955 | 1.100 (1.021, 1.185) 0.0123 |
| ≥90 | 723 | 1.190 (1.027, 1.378) 0.0205 |
| Physical Activity |  | 0.9778 |
| **Sedentary** | 1827 | 1.096 (0.973, 1.235) 0.1303 |
| **Light** | 3285 | 1.128 (1.018, 1.250) 0.0210 |
| Moderate | 2777 | 1.113 (0.992, 1.248) 0.0684 |
| High | 759 | 1.149 (0.885, 1.492) 0.2979 |
| Smoking |  | 0.7203 |
| No | 596 | 1.116 (1.040, 1.198) 0.0023 |
| Yes | 7533 | 1.075 (0.876, 1.318) 0.4901 |
| Drinking status |  | 0.6112 |
| Never | 598 | 1.137 (0.973, 1.329) 0.1054 |
| Current | 1386 | 1.035 (0.954, 1.101) 0.2075 |
| Ever | 6694 | 1.078 (1.021, 1.095) 0.0016 |

Note 1: Above model adjusted for age, BMI, drinking status, ALT, HDl-c, LDL-c, HAB1C, physical activity, DBP, smoking status, Scr, AST, hypertension, DLP-MED, and SBP.

Note 2: In each case, the model is not adjusted for the stratification variable.

HR, Hazard ratios; CI: confidence, Ref: reference.

**Table S3 Comparison of baseline characteristics between participants with** **TyG-GGT <380 and TyG-GGT ≥380**

| TyG-GGT quartile | <380 | ≥380 | P-value |
| --- | --- | --- | --- |
| N | 7152 | 1526 |  |
| Age(years) | 41.52 ± 8.66 | 43.03 ± 8.26 | <0.001 |
| SBP (mmHg) | 115.92 ± 12.18 | 121.70 ± 13.19 | <0.001 |
| DBP (mmHg) | 75.38 ± 7.97 | 79.27 ± 8.08 | <0.001 |
| BMI (kg/m^2^) | 25.65 ± 3.72 | 28.02 ± 4.04 | <0.001 |
| WC(cm) | 89.73 ± 12.01 | 97.72 ± 10.85 | <0.001 |
| TC (mg/dL) | 194.08 ± 35.66 | 211.53 ± 38.27 | <0.001 |
| LDL-c(mg/dL) | 120.99 ± 33.13 | 132.98 ± 34.75 | <0.001 |
| HDL-c(mg/dL) | 50.06 ± 13.41 | 44.21 ± 11.18 | <0.001 |
| HBA1C (%) | 4.64 ± 0.30 | 4.77 ± 0.31 | <0.001 |
| AST(u/L) | 27.77 ± 10.83 | 34.95 ± 11.44 | <0.001 |
| ALT(u/L) | 35.65 ± 15.05 | 55.22 ± 26.25 | <0.001 |
| CRP (mg/dL) | 2.27 ± 5.04 | 2.91 ± 4.93 | <0.001 |
| Sex (n, %) |  |  | <0.001 |
| Female | 2104 (29.42%) | 107 (7.01%) |  |
| male | 5048 (70.58%) | 1419 (92.99%) |  |
| HTN (n, %) | 624 (8.72%) | 244 (15.99%) | <0.001 |
| DLP (n, %) | 1795 (25.10%) | 570 (37.35%) | <0.001 |
| HTN-MED (n, %) | 610 (8.53%) | 251 (16.45%) | <0.001 |
| DLP-MED (n, %) | 545 (7.62%) | 212 (13.89%) | <0.001 |
| Smoking (n, %) | 532 (7.44%) | 138 (9.04%) | 0.033 |
| Physical Activity (n, %) |  |  | <0.001 |
| **Sedentary** | 1429 (19.98%) | 428 (28.05%) |  |
| **Light** | 2693 (37.65%) | 592 (38.79%) |  |
| Moderate | 2378 (33.25%) | 399 (26.15%) |  |
| High | 652 (9.12%) | 107 (7.01%) |  |

Continuous variables were presented either as mean ± standard deviation or as median with interquartile range , depending on data distribution. Categorical data were reported as counts and percentages. Abbreviations: Systolic blood pressure (SBP), diastolic blood pressure (DBP), body mass index (BMI), waist circumference (WC), total cholesterol (TC), low-density lipoprotein cholesterol (LDL-c), high-density lipoprotein cholesterol (HDL-c), Hemoglobin A1c(HbA1c), aspartate aminotransferase (AST), alanine aminotransferase (ALT), C-reactive protein (CRP), hypertension (HTN), antihypertensive medication (HTN-MED), dyslipidemia (DLP), antihyperlipidemic medication (DLP-MED).

Table S4. The relationship between TyG-GGT and the risk of DM in different sensitivity analysis

| Exposure | Model I (HR,95%CI) P | Model II (HR,95%CI) P |
| --- | --- | --- |
| TyG-GGT (per 50-unit) | 1.113 (1.038, 1.194) 0.003 | 1.103 (1.024, 1.189) 0.009 |
| TyG-GGT quartiles |  |  |
| Q1 | Ref | Ref |
| Q2 | 1.145 (0.718, 1.825) 0.571 | 1.142 (0.716, 1.823) 0.577 |
| Q3 | 1.460 (0.924, 2.308) 0.105 | 1.432 (0.900, 2.277) 0.130 |
| Q4 | 1.711 (1.076, 2.721) 0.023 | 1.619 (1.003, 2.614) 0.049 |
| P for trend | 1.205 (1.051, 1.380) 0.007 | 0.024 |

Abbreviations: TyG-GGT:the triglyceride-glucose-gamma-glutamyl transferase, Ref: reference; HR, Hazard ratios.

Model I is a sensitivity analysis in which the timing of DM occurrence is redefined as the midpoint between the date of the last normal physical examination and the date of the current abnormal physical examination (n=8678). HTN, DLP_MED, SBP, DBP, ALT, AST, HDL-c, LDL-c, HbA1c, Scr, physical activity, smoking status, drinking status, and age were adjusted.

Model II was a sensitivity analysis using complete case data (before multiple imputation; n=7,845). HTN, DLP_MED, SBP, DBP, ALT, AST, HDL-c, LDL-c, HbA1c, Scr, physical activity, smoking status, drinking status, and age were adjusted.

Table S5. Time-dependent ROC curves of baseline TyG-GGT for predicting incident DM within 1.0, 2.0, 3.0, 4.0, and 5.0 years.

| Follow-up(years) | N | events | AUC (95%CI) | Best threshold | Specificity | Sensitivity | Youden Index |
| --- | --- | --- | --- | --- | --- | --- | --- |
| 1.0 | 8497 | 181 | 0.7338(0.6935-0.7741) | 401.73 | 0.7403 | 0.6188 | **0.3591** |
| 2.0 | 8497 | 260 | 0.7292(0.6952-0.7631) | 398.50 | 0.7442 | 0.6107 | **0.3549** |
| 3.0 | 8497 | 280 | 0.7321(0.6997-0.7646) | 408.61 | 0.7451 | 0.6120 | **0.3571** |
| 4.0 | 8497 | 299 | 0.7342(0.7024-0.7660) | 409.59 | 0.8172 | 0.5375 | **0.3547** |
| 5.0 | 8497 | 310 | 0.7318(0.7000-0.7635) | 405.86 | 0.8173 | 0.5323 | **0.3496** |

Abbreviations: AUC: area under the curve; CI: Confidence interval.


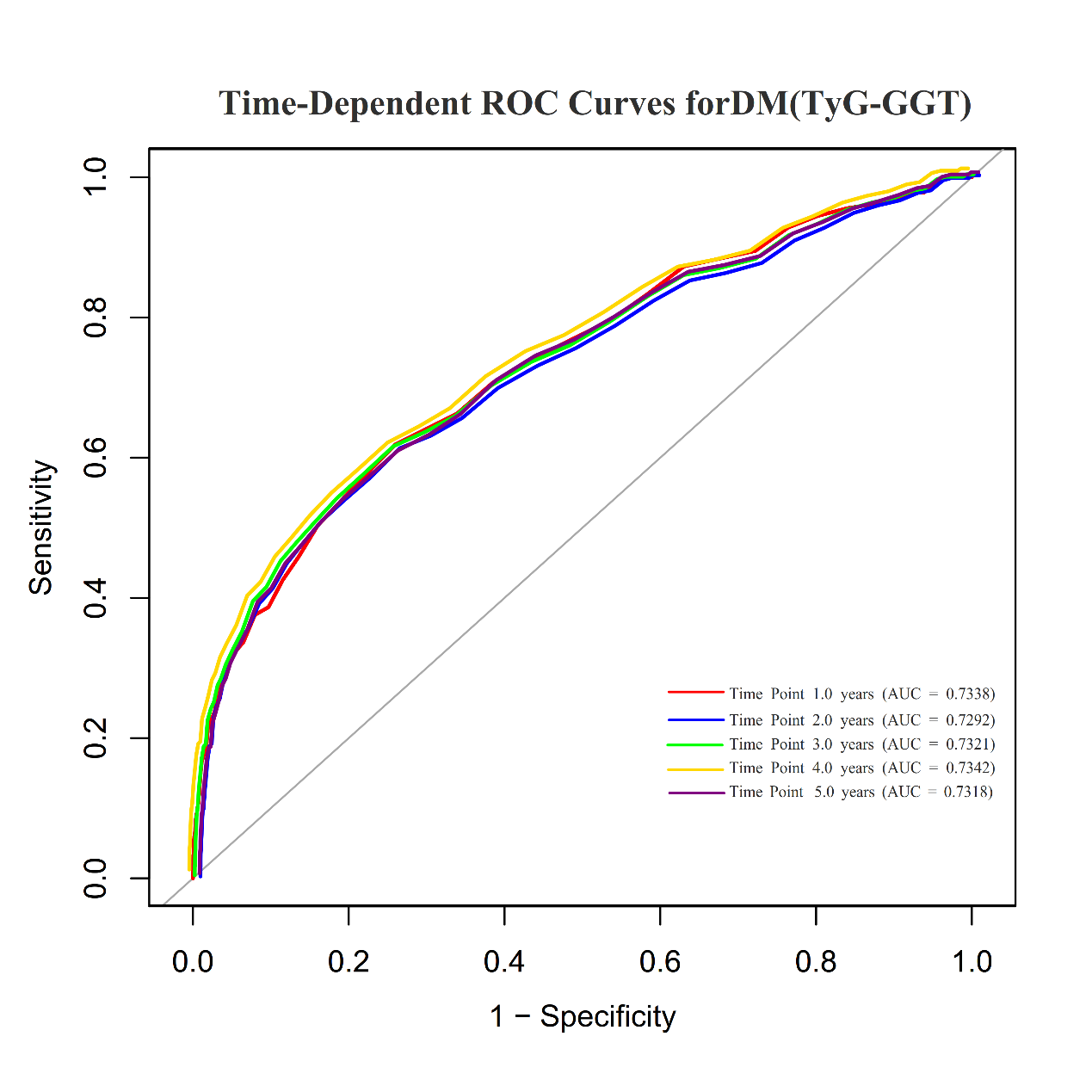


**Figure S1: Time-dependent ROC curves for TyG-GGT prediction of DM.**


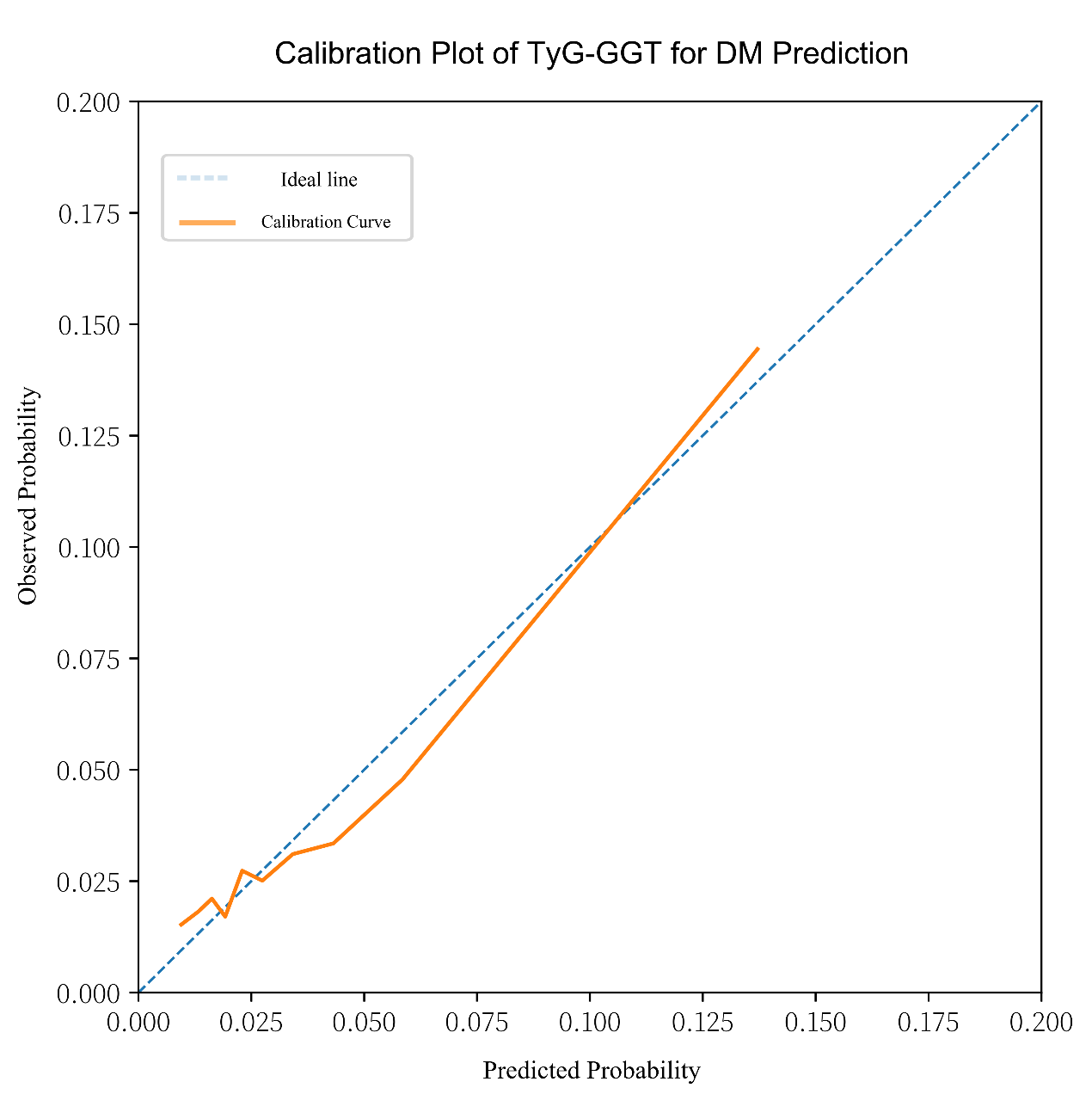


**Figure S2 Calibration curve for TyG-GGT in predicting DM**
